# Protein Unfolding and Aggregation near a Hydrophobic Interface

**DOI:** 10.3390/polym13010156

**Published:** 2021-01-03

**Authors:** David March, Valentino Bianco, Giancarlo Franzese

**Affiliations:** 1 Secció de Física Estadística i Interdisciplinària—Departament de Física de la Matèria Condensada, Facultat de Física, Universitat de Barcelona, Martí i Franquès 1, 08028 Barcelona, Spain; dmarchp@gmail.com; 2 Chemical Physics Department, Faculty of Chemistry, Universidad Complutense de Madrid, Plaza de las Ciencias, Ciudad Universitaria, 28040 Madrid, Spain

**Keywords:** protein, aggregation, unfolding, hydrophobic, coarse grain, Monte Carlo, simulation

## Abstract

The behavior of proteins near interfaces is relevant for biological and medical purposes. Previous results in bulk show that, when the protein concentration increases, the proteins unfold and, at higher concentrations, aggregate. Here, we study how the presence of a hydrophobic surface affects this course of events. To this goal, we use a coarse-grained model of proteins and study by simulations their folding and aggregation near an ideal hydrophobic surface in an aqueous environment by changing parameters such as temperature and hydrophobic strength, related, e.g., to ions concentration. We show that the hydrophobic surface, as well as the other parameters, affect both the protein unfolding and aggregation. We discuss the interpretation of these results and define future lines for further analysis, with their possible implications in neurodegenerative diseases.

## 1. Introduction

Proteins cover a range of fundamental actions in a living organism, including enzymatic and hormone functions, transport of biomolecules within the cellular environment, energy sourcing, tissues build and repair [1]. Typically a protein can perform these functions only when it is in its native and folded conformation.

Protein folding is a self-organized process occurring spontaneously in the aqueous solution, at least for small proteins, and it is dictated mostly by the protein sequence. After the synthesis at the ribosome, the polypeptide chain finds itself in a highly crowded cellular environment. Here, despite many non-specific interactions, the chain is capable of selecting a subset of amino acid contacts that funnel the free energy landscape toward a unique native/folded state.

However, the accumulation of partially folded conformations or the competition with other unfolded proteins could hinder the folding process, resulting in the formation of macromolecular aggregates [2]. Proteins can aggregate after they fold in the native state—through chemical bonding or self-assembling—or via unfolded intermediate conformations. In particular, non-native protein-aggregates are commonly formed through a multi-step process and are made of native-like–partially-folded intermediate structures [3,4,5,6].

Proteins have a propensity to aggregate related to a series of factors, e.g., the flexibility of the protein structure [7] or the sub-cellular volume where the protein resides [8]. They evolved toward a low aggregation-propensity, within a range of protein expression required for their bioactivity. However, they have no margin to respond to external factors that increase or decrease their expression or solubility [8,9,10]. As a consequence, inappropriate protein aggregation represents a crucial issue in biology and medicine. It is associated with a growing number of diseases, such as Alzheimer’s and Parkinson’s disease [11,12,13,14], and with the degradation of the pharmaceutical product quality and performance [15].

Among different strategies to tackle the related diseases, many hopes have been placed in using functionalized nanoparticles for inhibiting protein and peptide aggregation [16,17]. However, once in the bloodstream, the nanoparticles form the protein corona [18,19]. This corona can alter the biological effect of the nanoparticle and can induce unexpected reactions [20,21].

Many aspects of the nanoparticle interface, such as the shape, the size, or the surface chemistry, can affect the aggregation of proteins [22,23,24,25,26,27]. Nonetheless, the capability of proteins to keep their native conformation upon aggregation or adsorption onto inorganic interfaces is still poorly understood.

Computational approaches are gaining ground as fundamental tools to investigate these phenomena and the interplay between folding and aggregation in homogeneous and heterogeneous solutions of proteins. In particular, coarse-grain models and multiscale methods allow us to deal with such complex systems [28,29].

For example, a recent study showed that the concentration increase of individual protein species can unfold their native state without inducing their aggregation [30]. Furthermore, each component in a protein mixture can keep its folded state at densities that are larger than those at which they would precipitate if they were in a single-specie solution [31]. These works study a lattice model of proteins embedded in an explicit coarse-grain water-model. The models account for the protein features [32,33,34,35,36,37] and the water thermodynamics [38,39,40,41,42,43], representing a promising approach to study the behavior of protein solutions at an inorganic interface.

This approach has been exploited to better understand the mechanisms of cold and pressure denaturation of proteins and the effect of water-mediated interactions. Taking into account how water at the protein interface changes its hydrogen bond properties and its density fluctuations, the model can predict protein stability regions with elliptic shapes in the temperature-pressure plane, consistent with other theories and experiments, identifying the different mechanisms with which water participates to denaturation by changing temperature or pressure [35].

Furthermore, this model can be used to design proteins at extreme conditions of temperature and pressure. It has clarified that the limits of stability in temperature and pressure, and the selection mechanisms at extreme conditions, relate to the temperature and pressure dependence of the properties of the surrounding water [37]. As a consequence, the hydropathy profile of the proteins results from a selection process influenced by water, with superstable proteins at high temperatures characterized by nonextreme segregation between the hydrophilic surface and the hydrophobic core, while less-stable proteins have larger segregation or very low segregation [37].

Here, following the approach in Ref. [30,31], we present a computational study on the protein folding/unfolding and aggregation near a hydrophobic interface, representative of a portion of a nanomaterial. By performing Monte Carlo simulations, we describe the formation of aggregates against folded conformations at different temperatures, both in the bulk water and at the hydrophobic interface. We discuss the dependence of our finding on the water–water interaction in the protein hydration shell, linking the observed phenomena to the hydrophobic effect. Our results could shed light on the biological mechanisms underlying the formation of protein aggregates at the nanoscale.

## 2. Model

### 2.1. Franzese–Stanley Water Model

We adopt a coarse-grain representation of the water molecules, partitioning a volume *V* into a fixed number *N* of cells, each one with volume $v\equiv V/N\geq v_{0}$, with $v_{0}$ being the water excluded volume. For the sake of simplicity, we will consider here the case of the projection into two dimensions (2D) of a water monolayer with height h≃0.5 nm. Although a confined monolayer of water can have properties quite different from bulk water [44,45], here the dimensionality only affects the number of neighbors of each water molecule but does not change its coordination number (the number of hydrogen bonds formed by each water molecule). Indeed, regardless of whether the model is in 2D or 3D, each water molecule can form up to four hydrogen bonds. Our preliminary data show that this is sufficient to find no qualitative differences near ambient conditions between our water model in 2D and 3D [46].

We fix *T* and *P* of the system, leaving $r\equiv \sqrt{v/h}$ free to change, with $r\geq r_{0}$ the average distance between first neighbor water molecules. The model is able to describe all the fluid phases of water [42]. Here we focus only on its liquid phase. The Hamiltonian describing the interaction of the bulk water is

(1) $$H\equiv \sum_{ij}U\left(r_{ij}\right)-JN_{HB}^{\left(b\right)}-J_{\sigma }N_{\mathrm{coop}}^{\left(b\right)}.$$

The first term, summed over all the molecules *i* and *j* at oxygen–oxygen distance $r_{ij}$, accounts for the Van der Waals attraction and the repulsive forces due to Pauli’s exclusion principle, and is expressed as a double-truncated Lennard–Jones potential,

$$U\left(r\right)\equiv 4\epsilon \left[{\left(\frac{r_{0}}{r}\right)}^{12}-{\left(\frac{r_{0}}{r}\right)}^{6}\right],\;\;\mathrm{if}\;r_{0}<r<6r_{0},$$

U≡∞ for $r\leq r_{0}$ and U≡0 for $r\geq 6r_{0}$, where we use ϵ as our energy scale.

The second term of the Hamiltonian represents the directional (covalent) contribution to the formation of water–water hydrogen bonds (HBs) with characteristic energy *J*. Assuming that each molecule *i* can form up to four HBs, the number of possible molecular conformations is discretized by the introduction of four bonding variables $\sigma_{ij}=1,\cdots ,q$, one for each neighbor molecule *j*. Following a standard definition [47], two conditions must hold for the formation of a HB.

First, the molecules must be separated no further than $r_{\max}$. In a monolayer the condition $r<r_{\max}$ corresponds to $v/v_{0}<0.5$ for $v_{0}=r_{0}^{2}h$, with $r_{0}≃2.9$ Å van der Waals diameter of a water molecule, and $r_{\max}≃4$ Å. We associate to each water molecule *i* with a proper volume *v* an index $n_{i}=1$ if $v/v_{0}<0.5$, and $n_{i}=0$ otherwise. Hence, for the neighbor molecules *i* and *j*, the first necessary condition to form a HB is that $n_{i}n_{j}=1$.

Second, the angle $\hat{OOH}$ between two neighbor molecules must be less than ±30°. Therefore, only 1/6 of all the possible orientations [0°, 360°] are associated with a HB. Thus, we fix q=6, and the second condition to form a HB is that $\sigma_{ij}=\sigma_{ji}$, correctly accounting for the entropy loss associated with a HB formation. Therefore, the total number of bulk HBs is $N_{HB}^{\left(b\right)}\equiv \sum_{\langle ij\rangle }n_{i}n_{j}\delta_{\sigma_{ij},\sigma_{ji}}$, where $\delta_{a,b}=1$ if a=b, 0 otherwise, and the sum is over nearest neighbor molecules.

The third term of Equation (1) corresponds to the cooperative interaction of the HBs, emerging from quantum many-body interactions, which leads to an ordered, low-density tetrahedral configuration in bulk. This phenomenon is modeled as an effective interaction between each of the six different pairs of the four variables $\sigma_{ij}$ of a molecule *i*, coupled by an energy $J_{\sigma }$. $N_{\mathrm{coop}}^{\left(b\right)}\equiv \sum_{i}n_{i}\sum_{kl}\delta_{\sigma_{ik},\sigma_{il}}$ is the sum over the pair of bonding indices that cooperatively acquire the same value in each molecule *i*. By taking $J_{\sigma }\ll J$, we guarantee that the term plays a role only when the HBs are formed.

Finally, the total volume, and hence the density field, depends on the HB formation, as $V^{\left(b\right)}\equiv Nv+N_{HB}^{\left(b\right)}v_{HB}^{\left(b\right)}$, where $v_{HB}^{\left(b\right)}$ is a fraction of $v_{0}$. This relation accounts, on average, for the local decrease of density due to the tetrahedral HB network. The values of the model’s parameters are given at the end of the next section. Further details about the water model can be found in Ref. [48].

### 2.2. Protein and Interface Model

Following the coarse-grain representation for the water molecules, we adopt a coarse-grained lattice representation for the proteins, depicted as self-avoiding heteropolymers composed of 36 amino acids. For simplicity, each residue can occupy only one of the cells of the system.

The amino acids interact through the nearest neighbor potential given by the Miyazawa–Jerningan interaction matrix [49]. To account for the lower surface-volume ratio in 2D, we scale the matrix by a factor of 2, increasing the effective amino acids interactions [30].

Depending if two water molecules, forming a HB, are near two hydrophobic (Φ) amino acids, two hydrophilic (ζ) amino acids, or one of each kind (mixed, χ), the hydration-water Hamiltonian is

(2) $$H_{w,w}^{\left(h\right)}\equiv -\left[J^{\Phi }N_{HB}^{\Phi }+J^{\zeta }N_{HB}^{\zeta }+J^{\chi }N_{HB}^{\chi }\right]-\left[J_{\sigma }^{\Phi }N_{\mathrm{coop}}^{\Phi }+J_{\sigma }^{\zeta }N_{\mathrm{coop}}^{\zeta }+J_{\sigma }^{\chi }N_{\mathrm{coop}}^{\chi }\right],$$

where $N_{\alpha }^{\Phi }$, $N_{\alpha }^{\zeta }$, $N_{\alpha }^{\chi }$ (α=HB,coop) represent the number of directional and cooperative bonds formed at a hydrophobic, hydrophilic or mixed interface, respectively.

Experiments and simulations show that the water–water HBs near a hydrophobic interface are (i) stronger than bulk HBs, and (ii) increase the local water density upon pressurization [50]. To account for these effects, the model assumes that $J^{\Phi }>J$, $J_{\sigma }^{\Phi }>J_{\sigma }$, and that the volume associated with a HB at the Φ interface decreases upon a pressure *P* increment, i.e.,

(3) $$v_{HB}^{\Phi }/v_{HB,0}^{\Phi }\equiv 1-k_{1}P,$$

where $v_{HB,0}^{\Phi }$ is the volume increase for P=0, and $k_{1}$ is a factor accounting for the compressibility of the hydrophobic hydration shell. Thus, HBs in a hydrophobic hydration shell generate an extra contribution $V^{\Phi }\equiv N_{HB}^{\Phi }v_{HB}^{\Phi }$ to the total volume.

We adopt the simplified version of the model in which the HBs and the water density near a hydrophilic interface are as in bulk. The parameters for the HBs in the mixed, χ, case are an average between the Φ and the ζ case. Hence, the model sets $J^{\zeta }=J$, $J_{\sigma }^{\zeta }=J_{\sigma }$, $J^{\chi }=\left(J^{\Phi }+J^{\zeta }\right)/2$, $J_{\sigma }^{\chi }=\left(J_{\sigma }^{\Phi }+J_{\sigma }^{\zeta }\right)/2$, and lastly $v_{HB}^{\zeta }=v_{HB}^{\left(b\right)}$, where $v_{HB}^{\left(b\right)}$ is the bulk HB-volume parameter.

Finally, the model assumes that the protein–water isotropic interaction energy is different depending on the residue nature. In particular, it is $-\varepsilon^{\Phi }$ and $-\varepsilon^{\zeta }$ in the hydrophobic and the hydrophilic hydration shell, respectively.

### 2.3. The Hydrophobic Surface

We model the hydrophobic interface as a flat surface with excluded-volume interaction with both water and proteins. We fix it in space, separating our systems into two parts. Because we consider periodic boundary conditions (PBC), our system corresponds to an infinite volume confined between two parallel hydrophobic surfaces at a distance equal to the size *L* of the system. The water–water HBs near the hydrophobic interface are as in the Φ case described above.

### 2.4. The Model’s Parameters

Following Ref. [30], we choose model’s parameters that balance the water–water, water–residue, and residue–residue interactions, ensuring the protein stability in the liquid phase, including ambient conditions. Furthermore, by enhancing the interfacial interactions, they also account for the protein and interface surface loss by taking a 2D representation instead of 3D. As described in the following, we use three different sets of parameters to understand how our results depend on them. The three sets, here called Scale 0, 1, and 2, are indicated in Table 1. For the sake of comparison, Scale 0 is the same set of parameters adopted in Ref. [30].

## 3. Method

### 3.1. The Protein

**Figure 1:**
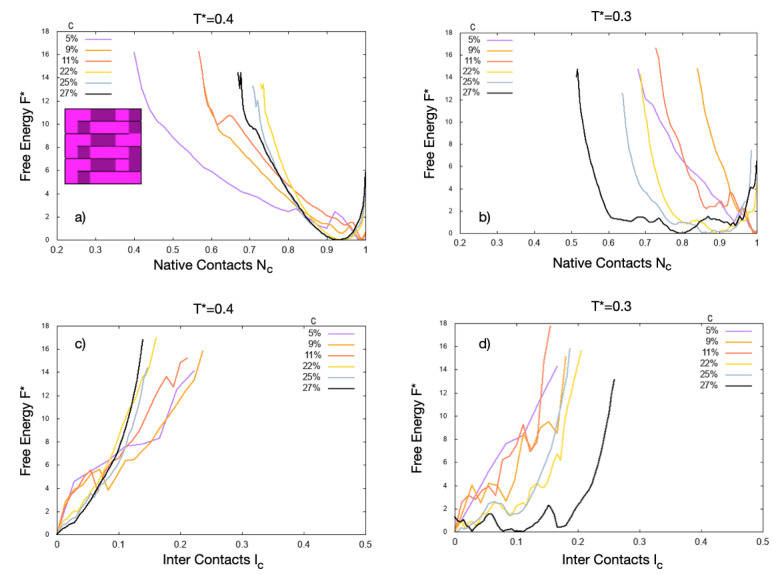
The *snake*-proteins free energy changes with concentration *c* and temperature $T^{*}$. The concentrations go from c=5% (violet) to 27% (black), as indicated in the legend. (**a**) Inset: Native structure of the *snake* protein; the dark/light cells are hydrophobic/hydrophilic amino acids. Main panels: $F^{*}\left(N_{c}\right)$ at warm temperature ($T^{*}=0.4$), and (**b**) at ambient temperature ($T^{*}=0.3$), has a minimum that moves from $N_{c}=1$ at low *c* to $N_{c}<1$ at high *c*, with a change between the folded and the unfolded state at 11%<c<22%, corresponding to $5\leq N_{p}\leq 10$ proteins in our system. The change is greater at ambient conditions. (**c**) For warm temperature and the same concentrations, $F^{*}\left(I_{c}\right)$ has a minimum always at $I_{c}=0$, showing that the proteins do not aggregate at this temperature, regardless if they are folded or unfolded. (**d**) The result is different at ambient conditions. The free energy develops several minima at the larger concentration, c=27% (12 proteins), showing that the unfolded proteins aggregate at high concentration. In all the panels, the curves at lower concentrations are noisier than those at higher *c* because the corresponding averages are over smaller numbers of proteins. Hence, the fluctuations along the curves are an indication of the error bar on the estimates. In general, a detailed study of the free-energy landscape to estimate the possible occurrence of free-energy barriers would imply much larger statistics, which is out of the scope of the present work.

We study proteins with a *snake*-like native state (Figure 1, a inset). For comparison, we choose the $A_{0}$ protein introduced in Ref [30], which is in its native state at ambient conditions. Each protein has 36 residues and a hydrated interface of 20 amino acids of which 7 (35%) are hydrophobic and 13 (65%) hydrophilic. It has one side fully hydrophilic and no side completely hydrophobic.

### 3.2. The Monte Carlo Simulation

We perform Monte Carlo (MC) simulations of *snake* proteins embedded into a square lattice, with size L=40 and PBC. We consider protein concentrations in the range c=[4.5%,27%] in volume, from $N_{p}=2$ to 12 proteins. We simulate the system at ambient conditions, as in Ref. [30], and at a warmer temperature. In internal units of the coarse-grained water model, these thermodynamic conditions correspond qualitatively to set $k_{B}T/\varepsilon =0.3$ for ambient conditions, and $k_{B}T/\varepsilon =0.4$ for warmer water, both at P=0.

Following Ref. [30], each MC step is defined by the following sub-steps:

1. We choose randomly a global protein-move among shift, rotation, crankshaft, or pivot [51]. Then, we pick at random one of the proteins, and we attempt the selected global move. We repeat the random selection for $N_{p}$ times, updating on average all the proteins.
2. We choose a random number *m* between 1 and $4L^{2}$. For *m* times, we select one of the $L^{2}$ cells. If it includes an amino acid, we attempt a corner flip, i.e., the local protein-move [51]. If it includes a water molecule, we select one of its four σ-variables and attempt to change its state, hence, breaking or forming a HB.
3. We attempt a global change of the system volume.

We accept or reject each step following the MC detailed-balance rules. This algorithm guarantees that, for each possible global change in the protein configurations, there is a random number of local moves for the proteins or the water. This choice allows the system to re-equilibrate during the process.

### 3.3. The Observables

To study the proteins folding/unfolding and aggregation, at each MC step, we calculate the number $N_{c}$ of native contacts of each protein, i.e., contacts in common with the native structure, normalized by its maximum value ($25N_{p}$). Furthermore, we compute the number $I_{c}$ of inter-contacts between different proteins, or between proteins and the interface, normalized by its maximum ($36N_{p}$). Finally, we calculate the number $M_{c}$ of contacts of the proteins with the interface, normalized by (2L).

For each *c* and *T*, first, we equilibrate the system. We start from a high-*T* configuration for water, where we distribute the proteins in a homogenous way in their extended configuration. We consider that the system is equilibrated when all the observables $N_{c}$, $I_{c}$, and $M_{c}$ at each MC step fluctuate around an average value without displaying any drift at least for $10^{6}$ MC steps. We observe that, for Scale 0 parameters (Table 1), $1\times 10^{6}÷10\times 10^{6}$ MC steps provide enough time to reach equilibrium, depending on *c* and *T*. The equilibration time is longer for larger *c* and smaller *T*. For Scale 1 and 2, which we studied only at concentration 11%, the system slows down, requiring $12\times 10^{6}$ MC steps of equilibration.

Once at equilibrium, we calculate during $10^{7}$ MC steps the probability of occurrence, P(O), for each observable *O*. Finally, we compute the free-energy as function of each observables, $F\left(O\right)\equiv -k_{B}T\ln P(O)$. In the following, we use dimensionless temperature $T^{*}\equiv k_{B}T/\varepsilon$ and free-energy $F^{*}\equiv F/\varepsilon$.

## 4. Results

### 4.1. Scale 0

We first simulate the system at different concentrations and warm temperature. The free energy as a function of the normalized number of native contacts, $F^{*}\left(N_{c}\right)$, shows a minimum near $N_{c}=1$ at low concentration, c≤11% (Figure 1a). In this regime, all the proteins fold in their native conformation.

At higher concentrations, c>11%, the minimum moves toward $N_{c}≃0.94$. Hence, the proteins are on average in slightly-unfolded states, with ∼94% of their native contacts.

The unfolding at c>11% is more evident at ambient temperature (Figure 1b). As the concentration increases, the $F^{*}$ minimum moves toward lower values of $N_{c}$ and becomes broader. Both changes indicate a larger propensity of the proteins to unfold toward configurations with less than 80% of their native contacts for increasing *c*.

We calculate, under the same conditions, the tendency of the proteins to aggregate or adsorb onto the hydrophobic surfaces. At the higher temperature, the proteins, on average, do not aggregate or adsorb at the concentrations considered here. This result is clearly shown by the free energy as a function of the normalized number of inter contacts, $F^{*}\left(I_{c}\right)$, with minima at $I_{c}^{*}=0$ at any *c* (Figure 1c).

However, the situation changes at ambient conditions (Figure 1d). By increasing the concentration, for c>25%, above the unfolding threshold c>11%, they aggregate or adsorb onto the hydrophobic walls, with a shallow minimum in $F^{*}\left(I_{c}\right)$ around $I_{c}≃0.1$ for c=27%. This minimum shows that, when the native contacts are less than 80%, more than 10% of the total volume of the proteins is aggregated or adsorbed at a high concentration. Further calculations clarify that this minimum is only due to protein–protein aggregation, with no contribution from surface-adsorbed proteins.

**Figure 2:**
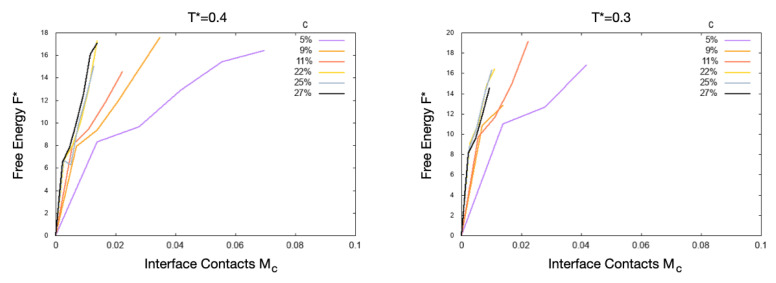
Free energy profiles as a function of the normalized number, $M_{c}$, of contacts of the proteins with the hydrophobic interfaces. Colors for the concentrations are as in Figure 1. The proteins are not adsorbed onto the interfaces when the minimum is at $M_{c}=0$.

Indeed, our direct evaluation of the free energy as a function of the normalized number of contacts of the proteins with the interface, $F^{*}\left(M_{c}\right)$, shows only minima at $M_{c}=0$ (Figure 2). Hence, for the specific sequence, *T*, and *c* we consider here, the surface-adsorption of the proteins would have a free energy cost that is too large for the system. This observation does not exclude that different sequences, with larger numbers of hydrophobic residues exposed to water in their native state, would adsorb at appropriate values of *T* and *c*.

**Figure 3:**
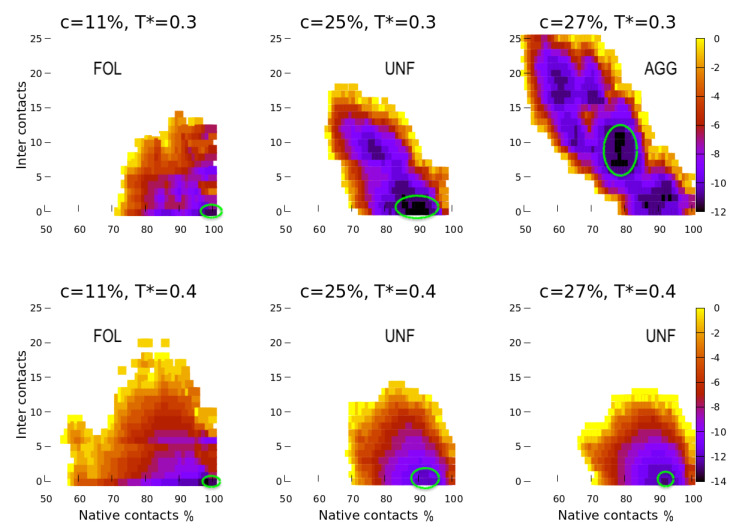
Color-coded free energy *F* as a function of the native contacts $N_{c}$ (horizontal axis, expressed as a percentage) and the average number of inter contacts $25N_{p}\times I_{c}$ (vertical axis), for different concentrations and temperatures. Darker colors correspond to deeper minima in free energy. We mark the absolute free-energy minima in green ellipses as guides for the eyes. We present data only for three concentrations. At ambient conditions (top panels) and c=11%, the proteins are mainly folded (FOL). At c=25%, they are unfolded (UNF) on average. At c=27%, they are unfolded and aggregated (AGG) in their majority. At the warmer temperature (bottom panels), they tend to avoid aggregation, even if they unfold. Statistically, where the volume concentration is low (11%), the proteins explore a larger number of configurations than at higher density, given the high thermal energy and the larger available volume.

Considering that $M_{c}$ is not affecting the free energy for our system in a significant way, we summarize our findings by the function $F^{*}\left(N_{c},I_{c}\right)$, showing how the free energy depends on the two relevant parameters $N_{c}$ and $I_{c}$ (Figure 3). We find a clear correlation between the two at ambient conditions and high concentration, with larger $I_{c}$ for smaller $N_{c}$. This result implies that at high concentration, c≥25%, the more the proteins unfold, the more they aggregate.

At lower *c* and higher *T*, this correlation is weak. In particular, $F^{*}\left(N_{c},I_{c}\right)$ at higher *T* (Figure 3, bottom) emphasizes the propensity of the proteins to unfold only partially at higher *c*, without aggregating. Interestingly, at the lower concentration in warm water, there is a larger probability of aggregation.

### 4.2. Scale 1 and Scale 2

Next, we explore how unfolding, aggregation, and surface-adsorption, depend on the competition between amino-acid interactions plus hydrophobic collapse, on the one hand, and entropy and energy-gain due to water–water HBs near hydrophobic interfaces, on the other hand. To this goal, we reduce the latter, by changing the values of $\varepsilon^{\Phi }$, $J^{\Phi }$, and $J_{\sigma }^{\Phi }$, as indicated in Table 1, with Scale 1 and Scale 2 sets of parameters compared to Scale 0.

**Figure 4:**
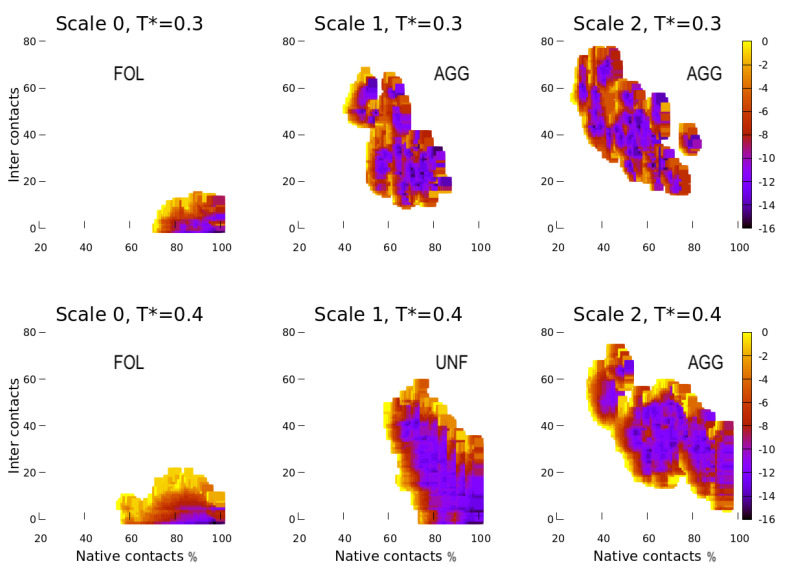
Comparing the free energy $F^{*}\left(N_{c},I_{c}\right)$ for different choices of model parameters, Scale 0, 1, 2 as indicated in Table 1, at fixed concentration c=11%, and ambient temperature (top row) or warmer temperature (bottom row). Axes and colors are as in Figure 3. Under these conditions, $F^{*}\left(N_{c},I_{c}\right)$ for Scale 0 (left) has a minimum corresponding to folded proteins (FOL). For Scale 1 (center), at ambient temperature (top), the proteins are unfolded and aggregated (AGG), while at the higher temperature (bottom), they unfold (UNF) but have a small tendency to aggregate. For Scale 2 (right), at both temperatures, the proteins aggregate (AGG) and are more unfolded at the lower temperature.

We fix the concentration at c=11% and simulate the system at both temperatures considered for Scale 0, with the parameters corresponding to Scale 1. We observe that the proteins with Scale 1 parameters (Figure 4, central panels) have a larger propensity to unfold and aggregate with respect to the model with the Scale 0 parameters (Figure 4, left panels). The effect is even more evident with Scale 2 parameters (Figure 4, right panels).

In particular, at warm temperature (Figure 4, bottom panels), the weakening of the HB parameters in the Φ-hydration shell increases the proteins’ propensity to unfold (Scale 1) and aggregate (Scale 2). At ambient temperature (Figure 4, top panels), both choices, Scale 1 and 2 lead to unfolded and aggregated proteins.

As a general trend, these calculations confirm that the aggregation propensity increases for the decreasing number of native contacts. Although the heterogeneity of the plots reveals the difficulty to explore the accessible configurations for Scale 1 and 2 and to locate the absolute free-energy minimum, especially at lower *T*, the overall trend of the results is clear. A more thorough calculation of $F^{*}\left(N_{c},I_{c}\right)$ is beyond the scope of the present work.

## 5. Discussion

### 5.1. Effect of the Hydrophobic Walls

To elucidate the effect of the hydrophobic walls, we compare our results, at ambient *T*, with those in bulk for the same protein [30]. We find that, at least for this specific protein ($A_{0}$ in Ref. [30]), the hydrophobic surfaces increase the concentration at which we observe unfolding, from $c_{\mathrm{FOL}\to \mathrm{UNF}}≃5\%$ to $11\%<c_{\mathrm{FOL}\to \mathrm{UNF}}^{S0}<22\%$, and the aggregation threshold, from $c_{\mathrm{UNF}\to \mathrm{AGG}}≃20\%$ to $25\%<c_{\mathrm{UNF}\to \mathrm{AGG}}^{S0}<27\%$, where quantities with the superscript S0 refer to the case presented here with the confining hydrophobic surfaces and Scale 0 parameters.

This result is apparently in contradiction with the findings in Ref. [31]. Bianco et al. show that proteins tend to fold uninfluenced by the presence of other proteins provided that their single concentration is below their specific $c_{\mathrm{FOL}\to \mathrm{UNF}}$ [31]. This result could suggest that even the presence of an additional interface, such as the hydrophobic surfaces considered here, would not affect $c_{\mathrm{FOL}\to \mathrm{UNF}}$.

However, there are fundamental differences between the cases considered in Ref. [31] and the present work. While in Bianco et al. [31], the additional interfaces (i) have a size comparable to the protein $A_{0}$, (ii) are heteropolymers made of different residues, and (iii) are fluctuating in their positions and configurations, here they are (i) made of two infinite walls, (ii) homogenous in their hydrophobicity, and (iii) fixed in space.

Hence, the confining walls here exclude a priori a number of protein configurations. On the other hand, the mixing with different proteins [31] alters the probability of some configurations for the protein $A_{0}$. However, it does not forbid any. Therefore, we suggest that our confinement pushes the FOL→UNF process toward protein concentrations that are higher than in bulk as a consequence of the limited ergodicity of the system.

While our confining walls induce a 120% increase in the FOL→UNF concentration with respect to the bulk case [30], our UNF→AGG concentration is only 25% larger than the value in bulk [30]. This result is consistent with the fact that now the proteins unfold at a higher concentration. Hence, the reduced free volume and the larger probability of protein–protein interaction partially compensate for the stabilization effect of the limited ergodicity.

In all the cases we considered here, we find that the proteins make very few contacts with the walls and do not adsorb onto them. We believe that this result is due mainly to the restrictions imposed by the 2D system, in which the interface is reduced just to a line of points.

Furthermore, the sequence we consider here is mostly (65%) hydrophilic. Hence, the proteins minimize their free energy when they are hydrated away from the walls.

We expect that proteins with larger hydrophobic patches would give rise to a more intense hydrophobic collapse, associated with the larger bulk-water entropy-gain and a stronger surface-adsorption. This investigation is underway.

More generally, it would be interesting to study how these results depend on the separation between the walls. For example, the system recovers the bulk case [31] at a large distance between the walls while at an intermediate distance, as the one considered here, both FOL→UNF and UNF→AGG move to higher protein concentrations, with no surface-adsorption. The question is if these processes can still occur at smaller distances and how they would change.

### 5.2. Effect of the Temperature

We observe that temperature affects both unfolding and aggregation. For the temperatures studied here, the effect is small for the unfolding, without leading to a change in the FOL→UNF threshold concentration. However, the protein at low *c* explores less unfolded states than at higher *T*, while they have a larger propensity to unfold at higher *c* and lower *T*. In any case, at lower *T*, the system equilibrates more slowly and has a larger statistical noise, especially at small *c*, because of the smaller number of proteins.

The effect is strong for the aggregation. While we find no aggregation at the warmer *T* up to c=27%, at ambient *T* it is $25\%<c_{\mathrm{UNF}\to \mathrm{AGG}}^{S0}<27\%$. We understand this result as a consequence of the larger propensity to unfold at lower *T* and higher *c*, and the thermal-energy decrease that favors the protein–protein interaction and aggregation.

Interestingly, at the higher *T*, the thermal energy hampers the aggregation of unfolded proteins, at large *c*, more than that of folded proteins, at small *c*. We interpret this finding as due to the larger steric hindrance of the loose ends of the unfolded proteins at high *T*.

### 5.3. Effect of the HB Strength near a Hydrophobic Interface

By going from Scale 0 to Scale 1 and 2, we reduce the HB strength near a hydrophobic interface. This weakening would correspond in an experiment, e.g., to an increase of ions concentrations in the protein solution and a decrease of the hydrophobic effect, which contributes to the stability of the folded state against *T* raises. Hence, at fixed *T* and *c*, the FOL state is less stable when we go from Scale 0 to Scale 1 and from Scale 1 to Scale 2. This observation implies that $c_{\mathrm{FOL}\to \mathrm{UNF}}^{S0}\geq c_{\mathrm{FOL}\to \mathrm{UNF}}^{S1}\geq c_{\mathrm{FOL}\to \mathrm{UNF}}^{S2}$, consistent with our results about the propensity to unfold at fixed concentration.

When we decrease the hydrophobic effect, the relative importance of the protein–protein interaction increases, favoring the AGG state. Hence, we expect $c_{\mathrm{UNF}\to \mathrm{AGG}}^{S0}\geq c_{\mathrm{UNF}\to \mathrm{AGG}}^{S1}\geq c_{\mathrm{UNF}\to \mathrm{AGG}}^{S2}$, consistent with our results.

As discussed for Scale 0, the aggregation is more prominent at lower *T* even for Scale 1 and 2. In particular, both Scale 1 and 2, at the chosen concentration c=11%, lead directly to the AGG state. Hence, for the set of parameters analyzed here, the $c_{\mathrm{UNF}\to \mathrm{AGG}}^{S}\left(T^{*}=0.3\right)<c_{\mathrm{UNF}\to \mathrm{AGG}}^{S}\left(T^{*}=0.4\right)$, where the superscript S refer to any of the scales for the parameters. Further analysis will be necessary to verify all the above relations in detail for different sets of parameters and temperatures, including both confined and bulk cases.

## 6. Conclusions

Following recent computational works, adopting the FS water model to study how folding/unfolding (FOL/UNF) compete with aggregation (AGG) when the protein concentration increases [30,31], here we consider the effect of nearby hydrophobic walls at different temperatures, concentrations, and hydrophobic strength.

In all these cases, we find that the aggregation is ruled by the unfolding. The more the proteins unfold, the more they aggregate. Increasing their concentration, the proteins first unfold, FOL→UNF, and next aggregate, UNF→AGG, with a range of concentrations for which the proteins unfold without aggregating, $c_{\mathrm{FOL}\to \mathrm{UNF}}<c_{\mathrm{UNF}\to \mathrm{AGG}}$ [30].

The presence of fixed hydrophobic walls increases both concentration thresholds at which the processes FOL→UNF and UNF→AGG occurr, i.e., $c_{\mathrm{FOL}\to \mathrm{UNF}}<c_{\mathrm{FOL}\to \mathrm{UNF}}^{S}$, and $c_{\mathrm{UNF}\to \mathrm{AGG}}<c_{\mathrm{UNF}\to \mathrm{AGG}}^{S}$, with a larger effect on FOL/UNF structural change.

We interpret these results as a consequence of the limitation of the accessible configurations that can be explored by the confined proteins (limited protein ergodicity). This effect is qualitatively different from what Bianco et al. observed in simulations and experiments for bi-component protein solutions [31], where protein–protein fluctuations alter the protein configurations probabilities but do not limit their ergodicity.

For the aggregation, the decrease in free volume at large *c* partially compensates the effect. For the chosen geometry and specific (*snake*) protein mainly hydrophilic, we do not observe surface-adsorption in the range of explored *T* and *c*. Further analysis is underway for more hydrophobic proteins.

Changes of *T* also affect the limiting concentrations of FOL, UNF, and AGG states. We find that at lower *T* the *snake* proteins have a larger propensity to unfold and a much stronger tendency to aggregate, as a consequence of the decreased thermal energy. In general, we find that $c_{\mathrm{UNF}\to \mathrm{AGG}}^{S}\left(T_{\mathrm{low}}\right)<c_{\mathrm{UNF}\to \mathrm{AGG}}^{S}\left(T_{\mathrm{high}}\right)$.

We find that changes in hydrophobic effect, as, e.g., due to an increase of ions in solution, also have a strong consequence on unfolding and aggregation. In particular, we consider three HB strengths near hydrophobic interfaces, high (S0), intermediate (S1), and small (S2), and find results consistent with $c_{\mathrm{FOL}\to \mathrm{UNF}}^{S0}\geq c_{\mathrm{FOL}\to \mathrm{UNF}}^{S1}\geq c_{\mathrm{FOL}\to \mathrm{UNF}}^{S2}$, and $c_{\mathrm{UNF}\to \mathrm{AGG}}^{S0}\geq c_{\mathrm{UNF}\to \mathrm{AGG}}^{S1}\geq c_{\mathrm{UNF}\to \mathrm{AGG}}^{S2}$. Hence, the decrease of the hydrophobic effect destabilizes the proteins against unfolding and aggregation at high concentrations, with a stronger repercussion at lower *T*.

Our results are potentially useful for the understanding of the mechanisms that control protein aggregation, a process that is associated with a growing number of neurodegenerative pathologies, including Alzheimer’s disease and Parkinson’s disease [52,53]. They represent the first step toward a multi-scale approach to study how to use nanostructured interfaces to regulate and, eventually, hamper pathological protein aggregation [54].

## Figures and Tables

**Table 1:** The three sets of parameters—here called Scales—considered in this work for the coarse-grained proteins, hydrated by the Franzese–Stanley water model. The three sets differ only for the values of $\varepsilon^{\Phi }$, $J^{\Phi }$, and $J_{\sigma }^{\Phi }$, associated with the water hydrating hydrophobic interfaces/residues. Symbols are defined in the text.

Scale	J/8ε	$J_{\sigma }/8\varepsilon$	$v_{\mathrm{HB}}^{\left(b\right)}/v_{0}$	$\varepsilon^{\Phi }/8\varepsilon$	$J^{\Phi }/8\varepsilon$	$J_{\sigma }^{\Phi }/8\varepsilon$	$v_{\mathrm{HB},0}^{\Phi }/v_{0}$	$k_{1}\varepsilon /v_{0}$	$\varepsilon^{\zeta }/8\varepsilon$
0	0.3	0.05	0.5	0.48	1.2	0.2	2	4	0
1	0.3	0.05	0.5	0.24	0.6	0.1	2	4	0
2	0.3	0.05	0.5	0.1	0.35	0.05	2	4	0

## Data Availability

The data that support the findings of this study are available from the corresponding author, G.F., upon reasonable request.

## Abbreviations

The following abbreviations are used in this manuscript:

AGG	Aggregated
FOL	Folded
HB	Hydrogen Bond
MC	Monte Carlo
PBC	Periodic Boundary Conditions
UNF	Unfolded
Φ	Hydrophobic
ζ	Hydrophilic
χ	Mixed
