# Correction: March et al. Protein Unfolding and Aggregation near a Hydrophobic Interface. *Polymers* 2021, *13*, 156

**DOI:** 10.3390/polym15092053

**Published:** 2023-04-26

**Authors:** David March, Valentino Bianco, Giancarlo Franzese

**Affiliations:** 1 Secció de Física Estadística i Interdisciplinària—Departament de Física de la Matèria Condensada, Facultat de Física, Universitat de Barcelona, Martí i Franquès 1, 08028 Barcelona, Spain; 2 Chemical Physics Department, Faculty of Chemistry, Universidad Complutense de Madrid, Plaza de las Ciencias, Ciudad Universitaria, 28040 Madrid, Spain

In the original publication [1], there was a mistake in Table 1 as published. The fifth column from the left (***ε^ϕ^*/8*ε***) was (0.48, 0.24, 0.1) and the last column from the left (***ε^ζ^*/8*ε***) was (0, 0, 0). The correct values should be (0, 0, 0) for the fifth column and (0.48, 0.24, 0.1) for the last one. In other words, the values from the fifth and last columns have been interchanged. The corrected Table 1 appears below. The authors state that the scientific conclusions are unaffected. This correction was approved by the Academic Editor. The original publication has also been updated.

## Figures and Tables

**Table 1:** The three sets of parameters—here called Scales—considered in this work for the coarse-grained proteins, hydrated by the Franzese–Stanley water model. The three sets differ only for the values of *ε*^Φ^, *J*^Φ^, and $J_{\sigma }^{\Phi }$, associated with the water hydrating hydrophobic interfaces/residues. Symbols are defined in the text.

Scale	*J*/8*ε*	*J_σ_*/8*ε*	$v_{HB}^{\left(b\right)}/v_{0}$	*ε*^Φ^/8ε	*J*^Φ^/8ε	$J_{\sigma }^{\Phi }/8\varepsilon$	$v_{HB,0}^{\Phi }/v_{0}$	*k*_1*ε*_/*v*_0_	ε^ζ^/8ε
0	0.3	0.05	0.5	0	1.2	0.2	2	4	0.48
1	0.3	0.05	0.5	0	0.6	0.1	2	4	0.24
2	0.3	0.05	0.5	0	0.35	0.05	2	4	0.1
